# Exercise- and Cold-Induced Human *PGC-1α* mRNA Isoform Specific Responses

**DOI:** 10.3390/ijerph17165740

**Published:** 2020-08-08

**Authors:** Camille Larson, Megan Opichka, Mark L. McGlynn, Christopher W. Collins, Dustin Slivka

**Affiliations:** School of Health and Kinesiology, University of Nebraska at Omaha, Omaha, NE 68182, USA; clarson@unomaha.edu (C.L.); mvandehei@mcw.edu (M.O.); markmcglynn@unomaha.edu (M.L.M.); christophercollins@unomaha.edu (C.W.C.)

**Keywords:** RT-qPCR, transcription, truncated, alternate promoter, primary promoter

## Abstract

Cold exposure in conjunction with aerobic exercise stimulates gene expression of *PGC-1α*, the master regulator of mitochondrial biogenesis. *PGC-1α* can be expressed as multiple isoforms due to alternative splicing mechanisms. Among these isoforms is *NT-PGC-1α*, which produces a truncated form of the *PGC-1α* protein, as well as isoforms derived from the first exon of the transcript, *PGC-1α-a*, *PGC-1α-b*, and *PGC-1α-c*. Relatively little is known about the individual responses of these isoforms to exercise and environmental temperature. Therefore, we determined the expression of *PGC-1α* isoforms following an acute bout of cycling in cold (C) and room temperature (RT) conditions. Nine male participants cycled for 1h at 65% Wmax at −2 °C and 20 °C. A muscle biopsy was taken from the vastus lateralis before and 3h post-exercise. RT-qPCR was used to analyze gene expression of *PGC-1α* isoforms. Gene expression of all *PGC-1α* isoforms increased due to the exercise intervention (*p* < 0.05). Exercise and cold exposure induced a greater increase in gene expression for total *PGC-1α* (*p* = 0.028) and its truncated isoform, *NT-PGC-1α* (*p* = 0.034), but there was no temperature-dependent response in the other *PGC-1α* isoforms measured. It appears that *NT-PGC-1α* may have a significant contribution to the reported alterations in the exercise- and temperature-induced *PGC-1α* response.

## 1. Introduction

Mitochondrial dysfunction is common in aging, obesity, and some metabolic and neurodegenerative diseases [1,2,3]. It can be caused by a disruption in the production (biogenesis) of novel mitochondria as well as the breakdown of unhealthy mitochondria. Mitochondrial biogenesis is largely controlled by the gene *PGC-1α*, known as the master regulator of the process [4]. Activation of mitochondrial biogenesis genes may be a way to enhance mitochondrial health in a patient suffering from mitochondrial dysfunction. Exercise plays a key role in the activation of this process. In order to further combat mitochondrial dysfunction, novel approaches that improve the exercise response are needed.

Aerobic exercise in a cold environment may be an effective means to accelerate exercise-induced mitochondrial development. More specifically, aerobic exercise paired with environmental cold exposure has been shown to upregulate the transcription of *PGC-1α* [5,6]. Some studies have not detected a statistically significant upregulation of *PGC-1α* in the cold compared to control conditions but did observe non-statistical effects [7,8,9,10]. Taken collectively, it appears that environmental cold interventions during aerobic exercise enhance the exercise-induced transcription of *PGC-1α* in humans. Studies differ in terms of subject characteristics, timing of cold interventions, and temperature of the cold condition. Additionally, these studies have all reported what appears to be data related to total *PGC-1α* mRNA. However, *PGC-1α* is transcribed and translated into various splice variants of the initial mRNA, known as isoforms. These isoforms have varying mechanisms and functions. It is important to consider which *PGC-1α* isoform is transcribed in order to interpret the applied potential of cold exposure at enhancing the exercise-induced *PGC-1α* response.

The existence of two promoters on the *PGC-1α* genes contributes to the number of isoforms that exist. The traditional canonical *PGC-1α* isoform (*PGC-1α-a*) is derived from the primary promoter, whereas other isoforms are derived from the alternate promoter. Transcripts from the alternate promoter can undergo different splicing events, leading to isoforms *PGC-1α-b* and *PGC-1α-c*. Furthermore, all these isoforms exist in a truncated form (*NT-PGC-1α*) that is produced by a splice between exons 6 and 7, cutting the transcript nearly in half [11]. These truncated isoforms appear to retain some of the function of full-length isoforms [12] but may play a larger role in angiogenesis than mitochondrial development [13]. Exercise robustly increases transcripts from the alternate promoter, but transcripts from the primary promoter are relatively unchanged at low and moderate intensity exercise and only increase in response to high intensity exercise [14,15]. The transcription of isoforms derived from the alternate promoter seems to be driven by ß-adrenergic receptor activation [16,17], while transcription from the primary promoter is stimulated by energy state/AMPK activation [18]. The exact role of each isoform is still under debate, and although there is likely overlap in the role of mitochondrial development, there also appears to be some specificity. According to a recent review, *PGC-1α-a* is mostly associated with mitochondrial biogenesis, *PGC-1α-b* is associated with cholesterol biosynthesis and the inflammatory processes, and *PGC-1α-c* is associated with cell cycle control and tissue remodeling [11]. Finally, the truncated (NT) versions of these isoforms (*NT-PGC-1α-a*, *NT-PGC-1α-b*, *NT-PGC-1α-c*) seem to have similar functions to their non-truncated counterparts [11]. The various mechanisms and actions of the *PGC-1α* transcripts highlight the need to investigate specific isoforms instead of simply measuring the total *PGC-1α* response. 

The purpose of this investigation was to detail the *PGC-1α* isoform specific response to aerobic exercise in a cold environment. This information will allow us to determine the molecular basis and practical implications of previous work detailing the total *PGC-1α* response with exercise in the cold [5,6,7,8,9,10]. If only investigating total *PGC-1α* response, specific isoform responses may be diluted, and important implications missed. These results highlight the need to further explore *PGC-1α,* specifically, the truncated isoforms. 

## 2. Materials and Methods 

Participants were informed of experimental procedures, risks, and benefits of their participation and all procedures were approved by the University of Nebraska Medical Center Institutional Review Board and conformed to the standards set by the Declaration of Helsinki. The data presented here is from a subset of participants that previously participated in a study describing the broader transcriptional response to exercise in the cold (i.e., did not identify specific *PGC-1α* isoforms) [9]. Participants were “recreationally active”, defined by engaging in physical activity for at least 3 days a week for the 3 months prior to the trial. Under ACSM’s stratification, all participants were considered “low risk” for exercise-related cardiac events.

### 2.1. Initial Testing

Nine college-aged males completed the exercise intervention as previously described [9], see Table 1. Briefly, preliminary testing consisted of anthropometric measurements and maximal exercise capacity. Body composition was measured using hydrostatic weighing correcting for estimated residual lung volume [19] and applying the Siri equation [20]. Maximal exercise capacity was measured on a cycle ergometer (Excalibur Sport, Lode, Groningen, The Netherlands) using a graded exercise protocol starting at 95 watts and increasing by 35 watts every 3 min until volitional fatigue. The highest measured oxygen consumption (Parvo Medics, Sandy, UT, USA) was considered VO_2_ peak. The workload associated with VO_2_ peak i.e., maximal wattage (Wmax), was calculated by adding the workload of the highest completed stage to the proportion of time in the final stage multiplied by the 35-watt stage increment. Workload for experimental trials was set at 65% Wmax.

### 2.2. Experimental Trials

Each participant completed two experimental trials in a randomized, counterbalanced cross-over design. Participants visited the lab following a 12 h fast and cycled for 1 h at 65% Wmax in a temperature and humidity control chamber (Darwin Chambers Company, St. Louis, MO, USA). One trial was conducted in cold conditions (C, −2 °C), and the other trial was conducted in room temperature conditions (RT, 20 °C). Trials were separated (1 week) for sufficient washout period [10]. Testing was done in July (average daily high temperature: 30.8 ± 0.5 °C) to limit the amount of cold acclimation. Participants were instructed to refrain from alcohol, caffeine, and exercise in the 24 h period prior to each experimental trial. Core temperature was taken with a rectal probe (RET-1, Physitemp Instruments, Clifton, NJ, USA) self-inserted by the subjects 12 cm past the anal sphincter. Skin temperature was measured with skin sensors (SST-2, Physitemp Instruments, Clifton, NJ, USA) placed on each subject’s chest and back. The temperatures of these two locations were averaged and reported as skin temperature. Core temperature, skin temperature, and heart rate (Polar Electronic, Kempele, Finland) were monitored continuously and averaged for the 1 h trial. The 3 h recovery period began immediately post-exercise, and participants were directed to remain fasted, avoid extreme temperature, and minimize activity. 

### 2.3. Muscle Biopsies

Muscle biopsies were taken from the vastus lateralis prior to exercise (Pre) and 3 h after exercise completion (Post). The 3 h recovery time point was selected because this is within the time course for peak *PGC-1α* transcription [21]. The biopsied leg was selected in a randomized, counterbalanced manner. Pre-biopsies were extracted ~10 cm proximal to the patella and ~5 cm lateral from the center of the thigh in the belly of the vastus lateralis. Post-biopsies were extracted from the same leg ~2 cm proximal to the pre-biopsy. Biopsies were performed using standard sterile techniques and a 5 mm Bergstrom needle with the aid of suction as previously described [9]. Excess blood, connective tissue, and fat were removed from the sample before the muscle was placed in a chemical stabilization agent (All-Protect, Qiagen; Hilden, North Rhine-Westphalia, Germany), and stored at 4 °C overnight before being transferred to −30 °C for additional analyses.

### 2.4. mRNA Extraction and cDNA Synthesis

Muscle samples (10–20 mg) were homogenized in 800 μL of Trizol (Invitrogen, Carlsbad, CA, USA) using an electric bullet blender homogenizer utilizing 1.5 mL Red RINO tubes (Next Advance Inc., Averill Park, NY, USA). Samples were then incubated at room temperature for five minutes, 160 μL of chloroform per 800 μL of Trizol was added, and the tubes were shaken by hand for 15 s. After another short incubation at room temperature (2–3 min), the samples were centrifuged at 12,000× *g* for 15 min. The aqueous phase was transferred to a fresh tube, and 400 μL of isopropyl alcohol was added and incubated overnight at −20 °C to further precipitate mRNA. The samples were then centrifuged at 12,000× *g* for 10 min at 4 °C, and the mRNA was washed by removing the supernatant and adding 800 μL of 75% ethanol. Samples were vortexed and then centrifuged at 7500× *g* for 5 min at 4 °C. The resulting RNA was quantified (average ± standard error, 219.2 ± 10.3 ng/µL) with a nano-spectrophotometer (Nanodrop, Thermo Fisher Scientific, MA, Wilmington, DE, USA). An Agilent 2100 Bioanalyzer (Agilent Technologies Inc, Santa Clara, CA, USA) was used to inspect the RNA quality (RIN: 9.2 ± 0.1). Finally, superscript-first-strand synthesis system for RT-qPCR (Superscript IV, Invitrogen, Carlsbad, CA, USA) was used to synthesize RNA to cDNA. Each sample within a subject was adjusted to contain a standard RNA concentration (3 ng/μL) by dilution using RNase free water. 

### 2.5. RT-qPCR

RT-qPCR was performed on the cDNA samples using SYBR green technology (SsoAdvanced Universal SYBR Green Supermix, Bio-Rad, Hercules, CA, USA) and primers designed specific to the amplified gene. Specificity of gene amplification was determined by analyzing the thermal dissociation curves. Each 20 μL reaction volume contained 10 μL SYBR Green Mastermix, 1 μL primers, 2.5 μL cDNA, and 6.5 μL water, with the exception of *PGC-1a-c* reactions, which required a lower concentration of primers to prevent primer-dimer annealing (10 μL SYBR Green Mastermix, 0.25 μL primers, 2.5 μL cDNA and 7.25 μL water). Samples were run using an Agilent AriaMX Real-Time PCR System (Agilent Technologies Inc., Santa Clara, CA, USA) running 1 cycle at 95 °C for 30 s, and 40 cycles of 95 °C for 5 s followed by 10 s at 60 °C. Quantification of mRNA for genes of interest (Total *PGC-1α*, Total *NT-PGC-1α*, *PGC-1α-a*, *PGC-1α-b*, and *PGC-1α-c*) used primer sequences previously reported [22] and obtained from Integrated DNA technologies (Coralville, IA, USA). See Table 2 for primer design specifics. Each run included a non-template control in triplicate. Fold-change was calculated using the 2^−ΔΔCT^ method [23] relative to stable reference genes and to the pre-intervention time-point. NormFinder software (MOMA, Department of Molecular Medicine, Aarhus University Hospital, Denmark) [24] was used to examine the stability of Glyceraldehyde-3 phosphate dehydrogenase (GAPDH), beta-2-microglobulin (B2M), and ribosomal protein S18 (RPS18) as reference genes. The stability value of the geometric mean of these genes was determined to be 0.122. Average coefficient of variation for the housekeeping gene triplicates was 1.8 ± 0.1%. In addition, a 2-way repeated measures ANOVA revealed no differences in the expression of the geometric mean of our three reference genes over time or between conditions (*p* > 0.05).

### 2.6. Statistical Analyses

Paired samples *t*-tests were used to determine differences between core temperature, skin temperature, sweat rate, and heart rate. Gene expression was analyzed using a 2 × 2 (time × condition) repeated measures ANOVA using SPSS (v.26, Chicago, IL, USA). If a significant F-ratio was detected, Fisher’s protected least significant difference was used to determine where differences occurred. A probability of type I error less than 5% was deemed significant (*p* < 0.05).

## 3. Results

### 3.1. Experimental Trials

As expected, chamber temperature was significantly different between RT and C conditions (*p* < 0.001) and humidity was similar (*p* = 0.176). The low chamber temperature in C allowed for lower skin temperature (*p* < 0.001), sweat rate (*p* = 0.010), and heart rate (*p* = 0.023) in the C compared to RT. However, due to the body’s thermoregulatory response during exercise, core temperature was slightly higher in the C than RT (*p* = 0.044), see Table 3.

### 3.2. Gene Expression

**Figure 1:**
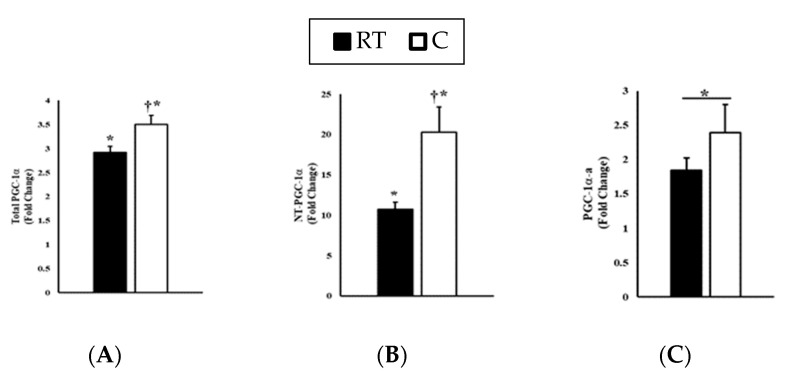

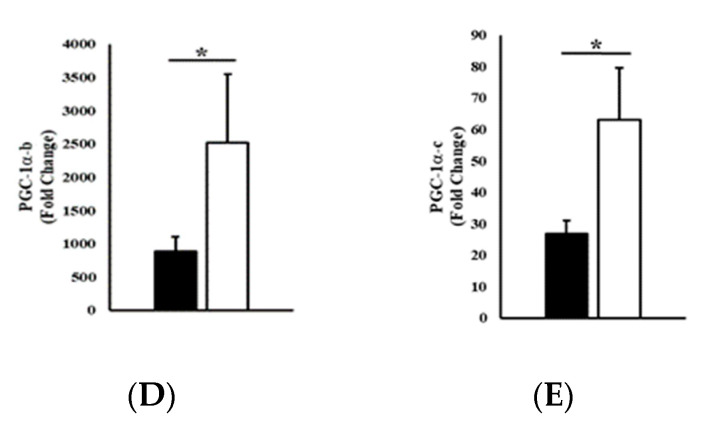
RT-qPCR analysis in response to exercise in cold (C, −2 °C) and in room temperature (RT, 20 °C) conditions. Fold-change was calculated using the 2^−ΔΔCT^ method [24] relative to stable reference genes and to the pre-intervention time-point. Thus, all pre-exercise values were normalized to 1.0. (**A**) Total *PGC-1α*; (**B**) Total *NT-PGC-1α*; (**C**) *PGC-1α-a*; (**D**) *PGC-1α-b*; (**E**) *PGC-1α-c*. Data are expressed as means ± SE (*n* = 9). * *p* < 0.05 from pre-exercise. † *p* < 0.05 from RT trial.

Total *PGC-1α* increased in response to exercise in both the RT and C trials (*p* < 0.001) but was higher in C than RT (*p* = 0.028; see Figure 1A) at 3 h post-exercise. Total *NT-PGC-1α* increased in response to exercise in both RT and C conditions (*p* < 0.001) but was higher in C than RT (*p* = 0.034; see Figure 1B) at 3 h post-exercise. *PGC-1α-a* increased in response to exercise (*p* = 0.003) but was not different between C and RT (*p* = 0.383; see Figure 1C). *PGC-1α-b* increased in response to exercise (*p* < 0.001) but was not different between C and RT (*p* = 0.340; see Figure 1D). *PGC-1a-c* increased in response to exercise (*p* < 0.001) but was not different between C and RT (*p* = 0.163; see Figure 1E).

## 4. Discussion

Exercise in cold environmental temperatures has been shown to enhance the exercise-induced response of *PGC-1α* [5,6]. The purpose of this investigation was to determine the isoform-specific responses of *PGC-1α* to aerobic exercise in a cold environment. The main finding of this investigation was that the truncated form of *PGC-1α* (*NT-PGC-1α*) appeared to be the major contributor to the overall *PGC-1α* response to exercise in the cold. Furthermore, cold did not statistically influence the expression of the other isoforms (*PGC-1α-a*, *PGC-1α-b*, and *PGC-1α-c*) after exercise. These data provide insight into the molecular responses related to exercise in a cold environment and may contribute to the understanding of the applied implications of cold enhanced *PGC-1α*.

Consistent with our previous environmental cold experimental protocols [6,8,9,10], average core temperature in the current study was slightly higher in the C than in the RT condition despite a lower skin temperature. This is a compensatory mechanism that overshoots in response to the cold skin temperatures. During cold exposure, the body preferentially vasoconstricts in the peripheries [25], thus conserving the metabolic heat generated in the core. This preferential vasoconstriction allows for enhanced venous return, and thus an increased stroke volume manifesting as a lower heart rate in the cold condition.

The similar structures of each isoform make the goal to target each isoform difficult. We took the approach of primer design that has been previously reported [15,22,26]. Because isolation of individual isoforms is unattainable using a single-primer PCR system, primers were designed to group structurally similar isoforms together. Total *PGC-1α* represents all isoforms as the primers span exons that are common to all isoforms. Total *NT-PGC-1α* primers spanned the gap between exons 6 and 7a, where a pre-mature stop codon is located and thus cannot differentiate between the different truncated isoforms (*NT-PGC-1α-a*, *NT-PGC-1α-b,* and *NT-PGC-1α-c*). Primers targeted the specific promoter regions but could not differentiate between the full-length and truncated isoforms. Therefore, *PGC-1α-a* also included *NT-PGC-1α-a*, *PGC-1α-b* also included *NT-PGC-1α-b*, and *PGC-1α-c* also included *NT-PGC-1α-c*. The application of this previously reported approach (i.e., Table 2) [15,22,26] differed from our laboratory’s previously used gene expression analysis [6,8,9,10], and, more importantly, was instrumental in allowing us to determine the specific splicing events that occurred within the *PGC-1α* complex. 

Total *PGC-1α* is the cumulative influence of all *PGC-1α* isoforms without regard to the specific promoter or other splicing events. Much of the data for *PGC-1α* in the literature is presumably total *PGC-1α*. We have previously reported that environmental cold enhances the exercise-induced response of total *PGC-1α* [5,6]. The current study confirms this observation, as total *PGC-1α* increases with exercise and is further enhanced when that exercise takes place in a cold environment. Total *PGC-1α* has been implicated in several intracellular pathways (calcium signaling, AMPK and MAPk signaling, ROS-mediated regulation, and ß-adrenergic signaling) [27]. The diversity of *PGC-1α* is likely achieved by the multiple isoforms and their specific responses. Much of the previous literature has neglected to investigate which isoforms are contributing to the total *PGC-1α* response and which pathways are preferentially targeted. Therefore, by exploring each isoform individually, we can better understand the true impact of *PGC-1α* and its many roles.

*PGC-1α-a* is derived from the primary promoter at exon 1a and has been associated with proteins that alter mitochondrial biogenesis and angiogenesis [17]. High-intensity exercise is required to activate *PGC-1α-a* via stimulation of AMPK [15,18]. The current study incorporated a 1 h cycling protocol at 65% of maximum power output. This robust exercise bout did enhance the transcription of *PGC-1α-a*, but cold did not appear to affect the exercise response. Thus, it appears the cold enhanced exercise response of total *PGC-1α* is not due to isoforms derived from the primary promoter.

These data suggest that isoforms derived from the alternate promoter of the gene contribute to the increase of *PGC-1α* seen after exercise more than isoforms from the primary promoter. Both *PCG-1α-b* and *PCG-1α-c* were robustly upregulated in response to exercise but were not statistically different in cold compared to room temperature exercise conditions. The large variability in response between subjects of these genes in the cold condition likely explains the lack of statistical difference, despite what appears to be a large difference, in the mean response. It is unclear what factors (fitness, body composition, acclimation, etc.) contributed to the large amount of variability. It does seem possible that cold may enhance the expression of *PCG-1α-b* and *PCG-1α-c,* since cold exposure leads to an increased catecholamine response thus stimulating ß-adrenergic receptors. Indeed, *PGC-1α-b* has been shown to increase in response to cold conditions in BAT of mice [18] and shift from the primary to alternate promoter [28]. Both *PCG-1α-b* and *PCG-1α-c* are related to mitochondrial biogenesis and fatty acid oxidation [14,17], but do also stimulate genes related to cholesterol biosynthesis, the inflammatory process, cell cycle control, and tissue remodeling [11]. Miura et al. [17] quantified the amount of mRNA from each isoform (relative to the amount of cDNA) and concluded that these alternate promoter isoforms contributed to only ~40% of the rise in total *PGC-1α* in mice skeletal muscle 3 h post-exercise. Previous works have suggested unidentified contributors to the overall *PGC-1α* response of a considerable percentage (40–50%) [17,29]. Based on these findings, and others [15,17,26,29,30,31], it appears these unidentified contributors may be the truncated forms of *PGC-1α* (*NT-PGC-1α*) and its truncated isoforms (*NT-PGC-1α-a, NT-PGC-1α-b,* and *NT-PGC-1α-c*).

These methods did not allow for analysis of each truncated isoform (*NT-PCG-1α-a*, *NT-PCG-1α-b*, and *NT-PCG-1α-c*), but rather to analyze the truncated forms collectively. Many of the same characteristics of the truncated forms are shared with their full-length counterparts [11,28], but some functions are unique. Total *NT-PGC-1α* appears to play a much greater role in angiogenesis than mitochondrial biogenesis [13]. *NT-PCG-1α-b* (also known as *PCG-1α-4*) appears to be strongly related to myogenesis and is more responsive to resistance exercise than aerobic exercise [30]. In the current study, we observed an exercise-induced increase in total *NT-PGC-1α*. Furthermore, exercise in the cold increased total *NT-PGC-1α* more than exercise in our control room temperature condition. It is interesting to note that other environmental extremes have also resulted in a preferential upregulation of *NT-PGC-1α* isoforms. Hypoxia has been implicated as a stimulus for post-transcriptional alternative splicing resulting in the upregulation of *NT-PGC-1α* spurring angiogenesis, yet only weakly inducing mitochondrial biogenesis [13]. Taken collectively, angiogenesis and the stimulators of *NT-PGC-1α* have a unique common denominator: the delivery of oxygen to muscle. Hypoxia, whether it be due to the demand of exercise, simulated hypoxia, the constriction of vessels to maintain an adequate temperature gradient, or any combination, seems to have a strong connection to the adaptive response of angiogenesis via the upregulation of *NT-PGC-1α.* These unique ambient and physiological phenomena speak to the complexity, sensitivity, and specificity of *PGC-1α* and all its truncated and non-truncated constituents. 

## 5. Conclusions

In conclusion, acute exercise in a cold environment appears to preferentially stimulate the alternate promoter, specifically the truncated form, *NT-PGC-1α*. This preferential stimulation may help explain why previous investigations, under similar conditions, were unable to reach statistical differences for total *PGC-1α*. More generally, this work supports previous data suggesting specific contributions to the overall *PGC-1α* among the isoforms, specifically the truncated isoforms enduring cold ambient temperatures. However, to what extent each isoform (full-length and truncated) may weigh in still needs clarification. Practically, future investigations should strive to further characterize, via absolute and relative abundance, the influence of temperature on the truncated forms of *PGC-1α* with the goal of optimizing novel approaches to improve the exercise response within diseases associated with dysfunctional mitochondria.

## Figures and Tables

**Table 1:** Participant Characteristics.

Variable	Participants
Age (y)	24.3 ± 5.7
Height (m)	179.4 ± 5.4
Weight (kg)	82.5 ± 13.9
Body Composition (%)	18.5 ± 6.2
VO_2_ peak (mL/kg/min)	45.6 ± 7.2

Note: Data are means ± SD (*n* = 9).

**Table 2:** Primer Sequences.

Transcript	Strand	Sequence
Total *PGC-1α*	FWD	AGCCTCTTTGCCCAGATCTT
	RVS	GGCAATCCGTCTTCATCCAC
Total *NT-PGC-1α*	FWD	TCACACCAAACCCACAGAGA
	RVS	CTGGAAGATATGGCACAT
*PGC-1α-a*	FWD	ATGGAGTGACATCGAGTGTGCT
	RVS	GAGTCCACCCAGAAAGCTGT
*PGC-1α-b*	FWD	CTATGGATTCAATTTTGAAATGTGC
	RVS	CTGATTGGTCACTGCACCAC
*PGC-1α-c*	FWD	TGAAAGTGAGTATCAGGAGGCA
	RVS	CTGATTGGTCACTGCACCAC
GAPDH	FWD	ACATCGCTCAGACACCATG
	RVS	TGTAGTTGAGGTCAATGAAGGG
B2M	FWD	GGACTGGTCTTTCTATCTCTTGT
	RVS	ACCTCCATGATGCTGCTTAC
RPS18	FWD	GTTCCAGCATATTTTGCGAGT
	RVS	GTCAATGTCTGCTTTCCTCAAC

Note: Based on Silvennoinen et al.’s work [22]. Primer sequences are from 5′ to 3′ direction; FWD: forward strand; RVS: reverse strand.

**Table 3:** Exercise Trial Data.

Variable	RT	C
Chamber Temperature (°C)	20.1 ± 0.2	−1.7 ± 1.5 *
Chamber Humidity (%)	66.8 ± 4.2	71.7 ± 6.6
Core Temperature (°C)	38.41 ± 0.48	39.04 ± 0.90 *
Skin Temperature (°C)	33.2 ± 1.4	28.1 ± 1.4 *
Sweat Rate (L/min)	0.86 ± 0.21	0.47 ± 0.38 *
HR (bpm)	160 ± 16	155 ± 13 *

Note: Data are means ± SD (*n* = 9). Values represent average of 1 h exercise bout. RT, room temperature; C, cold temperature; HR, heart rate. * *p* < 0.05 from Room Temperature.
